# Targeted Therapies in Liver Fibrosis: Combining the Best Parts of Platelet-Derived Growth Factor BB and Interferon Gamma

**DOI:** 10.3389/fmed.2015.00072

**Published:** 2015-10-05

**Authors:** Fransien van Dijk, Peter Olinga, Klaas Poelstra, Leonie Beljaars

**Affiliations:** ^1^Department of Pharmacokinetics, Toxicology and Targeting, Groningen Research Institute for Pharmacy, Groningen, Netherlands; ^2^Department of Pharmaceutical Technology and Biopharmacy, Groningen Research Institute for Pharmacy, Groningen, Netherlands

**Keywords:** liver fibrosis, drug targeting, therapy, PDGFβ receptor, PDGF-BB, IFNγ, hepatic stellate cell

## Abstract

Cytokines, growth factors, and other locally produced mediators play key roles in the regulation of disease progression. During liver fibrosis, these mediators orchestrate the balance between pro- and antifibrotic activities as exerted by the hepatic cells. Two important players in this respect are the profibrotic mediator platelet-derived growth factor BB (PDGF-BB) and the antifibrotic cytokine interferon gamma (IFNγ). PDGF-BB, produced by many resident and infiltrating cells, causes extensive proliferation, migration, and contraction of hepatic stellate cells (HSCs) and myofibroblasts. These cells are the extracellular matrix-producing hepatic cells and they highly express the PDGFβ receptor. On the other hand, IFNγ is produced by natural killer cells in fibrotic livers and is endowed with proinflammatory, antiviral, and antifibrotic activities. This cytokine attracted much attention as a possible therapeutic compound in fibrosis. However, clinical trials yielded disappointing results because of low efficacy and adverse effects, most likely related to the dual role of IFNγ in fibrosis. In our studies, we targeted the antifibrotic IFNγ to the liver myofibroblasts. For that, we altered the cell binding properties of IFNγ, by delivery of the IFNγ-nuclear localization sequence to the highly expressed PDGFβ receptor using a PDGFβ receptor recognizing peptide, thereby creating a construct referred to as “Fibroferon” (i.e., *fibro*blast-targeted inter*feron* γ). In recent years, we demonstrated that HSC-specific delivery of IFNγ increased its antifibrotic potency and improved its general safety profile *in vivo*, making Fibroferon highly suitable for the treatment of (fibrotic) diseases associated with elevated PDGFβ receptor expression. The present review summarizes the knowledge on these two key mediators, PDGF-BB and IFNγ, and outlines how we used this knowledge to create the cell-specific antifibrotic compound Fibroferon containing parts of both of these mediators.

## Introduction

Nowadays the most prevalent causes of chronic injury to the liver are chronic hepatitis B and C viral (HBV and HCV, respectively) infections, alcohol abuse, and metabolic problems related to obesity. Irrespective of the etiology of the disease, all types of chronic damage can finally lead to the induction of a common pathological response in the liver. In this common pathological response, the same cells and mediators play a role in the progression from chronic injury to fibrosis, cirrhosis, and eventually to tumor formation. At the moment, hepatocellular carcinoma is among the top three of most lethal carcinomas and its incidence is still increasing (1, 2).

Fibrogenesis is characterized by excessive fibrotic matrix deposition and by activation of macrophages and myofibroblasts. Within the liver, the major pool of matrix-producing myofibroblasts originates from activated and transformed hepatic stellate cells (HSCs), although other fibroblast sources such as the portal fibroblast or fibrocytes also have been identified (3–5). Clearly not only myofibroblasts and resident macrophages (Kupffer cells) are involved, but all other resident cell types contribute each in their own way to the fibrotic pathology. Additionally, infiltrating immune cells, both of the innate and acquired immune system, such as B cells, T cells, natural killer (NK) cells, NKT cells, neutrophils, and monocytes contribute to the disease progression. Their specific roles in the disease are becoming more and more clear (4, 6, 7). Although multiple cells are involved in hepatic fibrosis, crucial players are the fibroblasts, i.e., in the liver the activated HSCs and myofibroblasts, and macrophages, i.e., the Kupffer cells together with infiltrated monocytes. The fibroblasts are considered the master producers of all the fibrotic matrix proteins, whereas the macrophages are the master regulators (4, 5, 8).

Fibroblasts and macrophages communicate with each other via production and release of various growth factors, cytokines, and chemokines. The two most prominent profibrotic growth factors are platelet-derived growth factor BB (PDGF-BB) and transforming growth factor beta (TGFβ). While PDGF-BB stimulates fibroblast proliferation and migration, TGFβ is more involved in activation, transformation, and matrix production. In our studies, we chose the PDGFβ receptor (PDGFβR) as our target receptor for the delivery of antifibrotic compounds (9). The PDGFβR was preferred since the TGFβ receptor was not only expressed on HSCs and myofibroblasts but also hepatocytes and macrophages express this receptor (10–12). Next to the high production of profibrotic factors in diseased livers, various antifibrotic factors are produced and released as well (13). One of the major antifibrotic factors is interferon gamma (IFNγ). This cytokine is excreted by infiltrating T cells and NK(T) cells (6, 14). However, as disease progression continues, the antifibrotic factors cannot effectively counterbalance all profibrotic stimuli, leading to disturbance of the homeostatic balance and thus worsening of the disease.

In this review, we will give an overview of the characteristics and pathological roles of two mediators in fibrosis, i.e., PDGF and IFNγ, and summarize their receptor expressions and counterbalancing activities in various cell types. After that, recent studies on the structure, the receptor interaction, the *in vitro* and *in vivo* characteristics of the cell-specific therapeutic compound Fibroferon (previously called BipPB-PEG-IFNγ mimetic, mimγ-BipPB, or BOT191), which contains the most useful parts of both these abovementioned mediators, are summarized.

## Platelet-Derived Growth Factor

Platelet-derived growth factor, one of the first growth factors characterized, was discovered in the early 1970s as a key mediator involved in vascular pathologies such as atherosclerosis (15). Studies at that time proved that this growth factor, released from platelets, promoted vascular smooth muscle cell migration and proliferation. Later on, it became clear that PDGF was a potent mitogen for all mesenchymal cells, including fibroblasts and myofibroblasts. In later decades, PDGF was also identified as the most potent mitogen for HSCs in fibrotic livers (16). The distribution of the PDGF receptor correlated closely with the localization of activated mesenchymal cells predominantly present in collagen lesions within the liver (17).

Platelet-derived growth factor belongs to the large cysteine-knot superfamily. This family is characterized by the presence of eight conserved cysteine residues, forming a typical cysteine-knot structure. Within this large family, PDGF is structurally and functionally mostly related to the vascular endothelial growth factor (VEGF) family. The structure of the PDGF-family is highly conserved throughout the animal species (18, 19). Four different chains of PDGF-molecules are known: the traditional A- and B-chains and the more recently discovered C- and D-chains. The PDGF molecule is present in a dimeric form, and four homodimeric (AA, BB, CC, and DD) forms and one heterodimeric (AB) form are described. PDGF-A (211 aa, 24 kDa) and PDGF-B (241 aa, 27 kDa) are each other’s paralogs. Both proteins are processed mainly intracellularly and secreted in the dimeric active form. On the other hand, PDGF-C (345 aa, 39 kDa) and PDGF-D (370 aa, 43 kDa), also each other’s paralogs, are secreted in a dimeric latent form. These PDGF isoforms contain a so-called CUB (Complement C1r/C1s, Uegf, Bmp1) domain at the N-terminal region of these proteins. This CUB domain, mainly found in developmentally regulated extracellular and plasma membrane-associated proteins, causes retention of PDGF-CC and -DD in the extracellular matrix. For interaction with the PDGFR, extracellular activation of the C- and D-chain is necessary and it requires proteolytic cleavage of the CUB-domain by tissue plasminogen activator or urokinase plasminogen activator, respectively (18, 20). All five dimeric PDGF-forms can be found in the extracellular space but also in intracellular compartments, such as the endoplasmic reticulum and Golgi. There are even reports that state that the C-chain can be present in the nucleus (21).

Platelet-derived growth factor exerts its effects via binding to one of the two structurally related PDGF receptors, the PDGF alpha receptor (PDGFαR; Mw = 123 kDa) and beta receptor (PDGFβR; Mw = 124 kDa) that both belong to the class III receptor tyrosine kinases. Similar to the growth factor itself, also the receptor exists in homo (αα and ββ) and hetero (αβ) dimeric forms, all containing an extracellular part to which a particular PDGF-isoform binds and an intracellular signaling part. PDGF-A and C chains bind to the α-receptor, while B and D chains bind to the β-receptor. Both receptors are predominantly expressed on the cell membrane with minor expression in the lysosomes and even nucleus is reported (17, 19). In addition, PDGFαR was detected as a soluble receptor in the extracellular space (22).

### Biological effects

Platelet-derived growth factor has a clear role during embryonic development (18), but after that, in normal healthy tissues the activity and expression of the PDGF system is very limited. During pathological conditions, however, the PDGF system is activated again and diseases that are associated with overactivity of the PDGF system can be divided in three groups: tumors, vascular diseases, and fibrotic diseases. In fibrotic livers, several inflammatory and fibrotic mediators, such as interleukin-1β, tumor necrosis factor alpha (TNFα), TGFβ, and fibroblast growth factor (FGF), are released that can increase PDGF and its receptor expression. In addition, bacterial endotoxin (lipopolysaccharide), which is released in the serum after intestinal leakage and associated with liver fibrosis induction, can upregulate PDGFR expressions in the liver (23). The upregulation of the PDGFβ receptor in human cirrhotic livers was previously shown by Bansal et al. (24).

**Figure 1:**
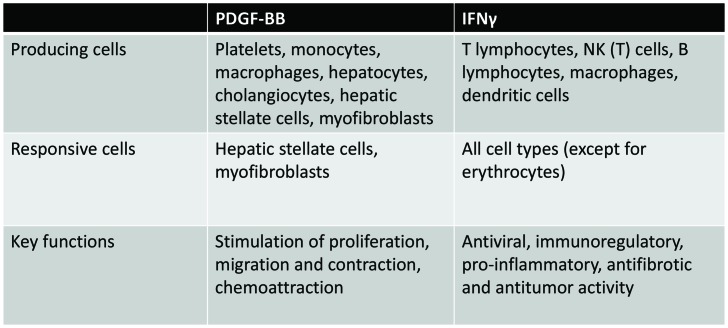
**Overview of PDGF-BB and IFNγ-producing cells and cells that respond to these mediators of fibrogenesis, illustrating the versatility of both mediators**.

Various isoforms of PDGF are reported to be present in the fibrotic liver, and all these isoforms have mitogenic and chemoattractive effects on mesenchymal cells. PDGF-BB is the major stimulus for mesenchymal cell proliferation, and the PDGFβ receptor is primarily involved in this cellular effect of PDGF. Also, activation of the β-receptor stimulates chemotaxis, whereas activation of the α-receptor is associated with either stimulation or inhibition of chemotaxis, depending on the mesenchymal cell type. PDGF is known for its effects on intracellular actin reorganization resulting in stimulated cell migration, chemotaxis, and contraction. There is, however, a difference between both PDGF receptors regarding their effects on the actin filament system. Whereas both receptors stimulate edge ruffling and loss of stress fibers, only the β-receptor mediates the formation of circular actin structures on the dorsal surface of the cell. Gu et al. nicely showed that these circular structures are involved in rapid recruitment, internalization, and membrane relocation of surface integrins, and in this way, these circular structures may serve as indicators of cellular transition from static to motile states (25). Together with TGFβ, PDGF-BB also induces fibroblast activation and differentiation, resulting in induction of α-smooth muscle actin (α-SMA) expression in these cells (19, 26). Figure 1 gives an overview of the PDGF-BB-producing and PDGF-BB-responsive cells and its key functions.

The PDGF isoform that is most prominent in liver fibrosis is PDGF-BB (16, 17). PDGF-BB is secreted by both resident and infiltrating cells of the liver, such as platelets, infiltrating monocytes and macrophages, activated Kupffer cells (M2-dominant type), hepatocytes, cholangiocytes, HSCs, and myofibroblasts, and it affects primarily HSCs and myofibroblasts. These latter cells display a highly induced PDGFβ receptor expression in mice and humans, and this expression can be related to the proliferative and migratory activities of these fibroblasts in fibrosis. Of note, activated HSCs and myofibroblasts produce PDGF-BB and thus create an autocrine profibrotic loop without interference of other cells.

Although PDGF production increases profoundly during the disease and thus local concentrations are high, the amounts present in the circulation are generally very low. While concentrations of only 17.5 ± 3.1 ng/ml were detectable in whole human blood, the amount in plasma was undetectable (27). The low concentrations in the circulation were due to a very short plasma half-life of less than 2 min and due to a high binding affinity to several proteins in plasma and extracellular spaces (27). Examples of PDGF-binding proteins are its soluble receptor, α2 macroglobulin, and PDGF-associated protein (PAP). Additionally, various extracellular matrix proteins are able to bind PDGF to form a local storage pool. This implies that the biological activities of PDGF can be modulated by binding to one of these molecules (28, 29).

### PDGF-receptor expression

Platelet-derived growth factor α-receptor and PDGFβR have overlapping, but also distinct, expression patterns and physiological roles. Generally, PDGFαR signaling is important in embryonic development since it controls gastrulation and the embryonic development of several organs such as lung, intestine, skin, testis, kidneys, bones, and neuroprotective tissues. Some cell types only express the PDGFαR, such as platelets and liver sinusoidal endothelial cells. PDGFβR signaling is recognized as an essential regulator of early hematopoiesis and blood vessel formation. The classical target cells for PDGF, fibroblasts and smooth muscle cells, express both α- and β-receptors, although the expression of the PDGFβR is generally at a much higher level. In fibrotic livers, PDGFβR is mainly expressed on the cell membrane of activated and transformed HSCs or myofibroblasts in the collagenous fibrotic bands (17, 24, 30). Borkham-Kamphorst et al. showed that the PDGFRs in the endothelium and hepatocytes are more of the α-type (30). In addition, PDGFαR was stained outside the area of CCl_4_-induced hepatocellular fibrosis, and they speculated that this increased expression could be related to hepatocyte survival. Furthermore, they found that the upregulation of PDGFαR was accompanied by PDGF-A and PDGF-C staining in regenerative hepatocytes after CCl_4_ intoxication.

How PDGF exactly binds to its receptor is the subject of various studies. Insight into this interaction can lead to the generation of PDGF receptor antagonizing drugs or to drug targeting compounds. Already in early 1990s, Clements et al. identified arginine 27 and isoleucine 30 as the PDGF-B chain residues that mediate the binding of the growth factor to the β-receptor in rat and human cells (31). Binding to the α-receptor requires another more cationic part of the molecule (arginine159-lysine160-lysine161-lysine162), and both in the PDGF-A and -B chain this sequence is present (32). More recently, the interaction between the growth factor and the receptor was studied in more detail with modern techniques like crystallography, partly confirming the abovementioned regions in PDGF, but also pointing to additional points of interaction with its receptors (33, 34).

### PDGF-receptor signaling

After binding of PDGF to one of the receptor chains, dimerization of the receptor is induced and this receptor dimer is then stabilized by the ligand. The subsequent interaction between the cytoplasmic domains of the two receptor chains induces phosphorylation of intracellular tyrosines leading to conformational changes in the PDGF receptor protein and thus to activation of the kinase domains of the receptor. Activation of src-homology-2 (SH2) or phosphotyrosine-binding (PTB) domains will lead to activation of one of the downstream cytoplasmic pathways. Although more pathways are described, activation of the MAPK/ERK pathway, the AKT/PKB pathway, and the PKC pathway are the major routes (19, 35). The MAPK/ERK pathway is also known as the Ras–Raf–MEK–ERK pathway because the GTP-binding protein Ras can activate mitogen-activated protein kinases (MAPK) such as extracellular signal-regulated kinase (ERK). Phosphorylated ERK-1 and -2 form activated ERK dimers and they translocate to the nucleus, where they can activate cellular processes linked to stimulation of cell proliferation and chemotaxis. Phosphoinositol 3-kinase can also be recruited to the activated PDGF receptor leading to stimulation of cell proliferation and migration via the AKT/PKB pathway. AKT, however, regulates multiple other biological processes including glycogen metabolism and cell survival, and it is linked to promotion of tumor growth. Activation of the third pathway involves phospholipase C-γ (PLC) recruitment after stimulation of the PDGFR. This causes mobilization of cellular Ca^2+^ storage pools and leads to activation of the intracellular signaling molecule protein kinase C (PKC). This route is also associated with increased cell growth and motility.

### Intracellular routing of PDGF and its receptor

Much is known about the intracellular signaling and biological effects of PDGFR. However, the cellular kinetics of the PDGFR itself is less well studied and also the fate of the growth factor and its receptor after binding of the ligand at the cell membrane leading to induction of intracellular signaling is poorly described. It is described that the PDGFβR is localized in specialized structures of the cell membrane referred to as caveolae (36–38). The morphology of these structures is generated by the protein caveolin and they are enriched in glycosphingolipids and cholesterol (39). Sorkin et al. showed that induction of the tyrosine kinase activity of the PDGFβR promotes ligand-induced internalization and subsequently degradation of the ligand–receptor complex in endosomal and lysosomal compartments of the cell. They showed that the receptors began to migrate out of caveolae after 1 h of exposure to PDGF and that the internalized PDGFβR retained kinase activity and thus could participate in signaling considerable time after internalization (36). The receptor can recycle back to the cell membrane waiting for its next signal. The fate of the growth factor after dissociation from the receptor in the endosomes is degradation in the lysosomal compartment, but other intracellular routes are possible (40).

## Interferon Gamma

The involvement of interferons (IFNs) in the defense response against viral infections was first described in 1957. Isaacs and Lindenmann reported that influenza virus-infected cells produced a secreted factor, i.e., interferon, that caused a virus-resistant state in previously uninfected cells (41). The biochemical characterization of this activity was pursued for a long time without success, and it was only in the late 1970s that the interferon protein sequence was ascertained (42, 43). Later, it appeared that the biological activities of IFNs were much broader, and antifibrotic, antitumor, immunoregulatory, and proinflammatory activities were also assigned to the IFN family (44).

Interferons, belonging to the interferon/interleukin-10 cytokine family, can be divided in two groups (type I and II). They are classified according to their receptor specificity and sequence homology (14). The type I IFNs, also described as viral IFNs, include IFNα (of which currently 17 subtypes are described), IFNβ, IFNω, and IFNτ. These type I factors are produced by almost all cell types. The type II IFNs consist only of IFNγ, which shows structurally little to no homology with type I IFN, and this protein is also described as immune IFN (45). In contrast to these type I IFNs, expression and secretion of IFNγ is not the result of the infection itself, but occurs by activated immune cells after recognition of infected cells (46). Therefore, although IFNγ possesses antiviral activity, it functions primarily as an immune modulator responsible for pathogen clearance rather than an antiviral compound (47, 48).

Interferon-γ is a non-covalently bound, homodimeric glycoprotein with subunits consisting of 146 amino acids (17.1 kDa) (49, 50), which are connected in an antiparallel fashion (45). Intracellular processing of this cytokine includes intensive glycosylation, giving rise to a mature form of the molecule that exhibits a predominant molecular mass of 50 kDa (51). Murine and human IFNγ show approximately only 40% sequence homology at the protein level (http://www.uniprot.org). The main cell types that produce IFNγ upon endogenous or exogenous stimulation in the fibrotic liver include T lymphocytes and NK(T) cells, B cells, and other antigen-presenting cells (52–58). NK cells were shown to be involved in the resolution of fibrosis in both mice and humans by stimulating the production of IFNγ, but also via killing of activated HSCs (59–61). The production of IFNγ in turn increased the number of NK cells (60). Emerging evidence suggests that NK cells and IFNγ are very important in the negative regulation of liver fibrosis (6). This was demonstrated by the amelioration of liver fibrosis *in vivo*, after activation of NK cells by poly I:C or treatment of rodents with IFNγ (60, 62–64).

### Biological effects

Within the liver, IFNγ exerts a variety of sometimes even opposing effects. For example, IFNγ is known to be a potent activator of macrophages, by stimulating them to kill phagocytosed microbes and cancer cells and inducing the production of TNFα and interleukin-12 (65). IFNγ induces an inflammatory (“M1”) activation state of the hepatic macrophages (comprising resident Kupffer cells and infiltrating monocytes and macrophages) (66). The proinflammatory factors produced via this route can activate the HSC to become a profibrotic myofibroblast. In addition, IFNγ induces the expression of both MHC-I and MHC-II in several cells (44), promotes the differentiation of T- and B-lymphocytes, and activates neutrophils, all contributing to promotion of fibrosis. It was also reported that IFNγ inhibits the proliferation of vascular endothelial cells (65). On the other hand, IFNγ has direct antifibrotic effects when it interacts with HSCs. Increased production of IFNγ, as secreted by NK cells in fibrotic livers, is accompanied by an increase in TNF-related apoptosis-inducing ligand (TRAIL) expression in NK cells, which causes both induction of apoptosis and cell cycle arrest in HSCs (60, 64, 67). The interaction between the stimulatory receptor NKG2D on NK cells and its ligand retinoic acid early inducible 1 (RAE1), expressed on activated HSCs, leads to killing of activated HSCs by NK cells and thus to resolution of fibrosis (60). A summary of cells that produce and respond to IFNγ and its main functions is presented in Figure 1.

In addition, IFNγ leads to reduced α-SMA, HSC proliferation, and collagen levels in fibroblasts (24, 62). IFNγ antagonizes the profibrotic activities of TGFβ, most probably by intracellular induction of the expression of the antagonistic Smad7. Smad7 causes impairment of the TGFβ response, via blocking of the interaction of the transcription factor Smad3 with the TGFβ receptor (68).

### IFNγ-receptor expression

Like many other cytokines, the receptor for IFNγ consists of multiple distinct subunits: the α chain (IFNγR1; 54.4 kDa), necessary for ligand binding and processing, and the β subunit (IFNγR2; 37.8 kDa), needed to induce a biological response by triggering the intracellular signal transduction cascade (14, 69). The complete receptor complex contains two IFNγR1 and two IFNγR2 subunits and these four parts associate after ligand binding. In more detail, IFNγ binds to one IFNγR1 and induces rapid dimerization of two IFNγR1 chains, thereby forming a site that is recognized by the extracellular domain of IFNγR2 (45). The IFNγR2 chain is generally the limiting factor in IFNγ responsiveness, as the IFNγR1 chain is usually in excess (45, 70). The IFNγR2 chain is constitutively expressed, but its expression level is tightly regulated according to the state of cellular differentiation or activation. Of note, the binding of human and murine IFN to their receptors is strictly species specific. They only induce an effect in species-matched cells. Moreover, the interaction of IFNγ with its receptor is not inhibited by type I IFNs (45).

Interferon-γ R1 and R2 are ubiquitously expressed on the cell membrane of virtual all types of cells, but most predominantly on cells of the immune system. In particular, macrophages are strongly positive for the IFNγR. Within the fibrotic liver, expression on infiltrated macrophages and Kupffer cells is high, while other cells like HSCs and hepatocytes weakly express the receptor (71). Bansal et al. previously showed the ubiquitous and increased expression of the IFNγR1 in human cirrhotic livers as compared to healthy livers (72). Although expression of the IFNγR at the plasma membrane varies widely between tissues (200–25,000 sites/cell), no direct correlation between the expression level and the magnitude of IFN-induced responses in cells was found (45).

### IFNγ-receptor signaling: The classical model

In the case of IFNγ, a conformational change in the receptor cytoplasmic domain is induced upon binding of IFNγ to the extracellular domains of two IFNγR1 chains. This causes movement of the inactive Janus activated kinase 2 (Jak2) from IFNγR2 to IFNγR1 and subsequent autophosphorylation and activation (73). After transphosphorylation and activation of Jak1 by Jak2, the recruitment of signal transducer and activator of transcription 1 alpha (Stat1α) is induced by phosphorylation of tyrosine440 residues of both IFNγR1 subunits, forming a homodimeric complex of pStat1α (69, 74–76). This Stat1 pair dissociates from the receptor upon phosphorylation at the tyrosine 701 residue, followed by active nuclear translocation via its nuclear localization sequence (NLS) (73, 74). In the nucleus, dimeric Stat1α binds to the IFNγ-activated sequence (GAS element) of the IFNγ-promoter, thereby determining specific gene activation of fibrosis-related genes like procollagen I and III and TGFβ, but also transcription factors like interferon regulatory factor-1 (IRF-1) (77–79). The cellular response of IFNγ occurs within 15–30 min after treatment (80). Binding of dimeric Stat1α to the GAS element is regarded as validation of the classical model of cytokine signaling (81).

To a lesser extent, IFNγ-signaling also produces heterodimeric complexes like Stat1:Stat2 and heterotrimeric complexes such as Stat1:Stat1:IRF-9 and Stat1:Stat2:IRF-9 (77, 82–84). These complexes are able to bind to IFN-stimulated response element (ISRE) promoter regions in target genes to regulate transcription of, for example, inducible nitric oxide synthase (iNOS), IRF-2, and IFNβ (14).

### IFNγ-receptor signaling: The non-canonical model

Several reasons led to the recognition that the classical model of cytokine signaling is an oversimplified model. It was shown that upon IFNγ stimulation besides the Jak/Stat pathway, also other pathways were activated or interfered with the Jak/Stat signaling. Examples include the activation of MAPK, phosphoinositide 3-kinase, Ca^2+^/calmodulin kinase II, and nuclear factor-κB (85). Moreover, it was shown in multiple cell lines that besides activated Stat, also Jak kinases were involved in the epigenetics of gene activation, indicating that Stat1 is not the only key player in the IFNγ signaling (86). In addition to this, many ligands activate the same Stat transcription factors, but have completely different effects in cells, tissues, or organs (87). In the classical model of signaling of IFNγ, it is suggested that the Stat proteins possess intrinsic NLSs, which are responsible for the nuclear translocation of Stat1 and subsequent IFNγ-related gene activation (88). Of note, in this classical model, the internalization of the ligand–receptor complex does not play an active role in the signaling process (87). Different to what was assumed in the classical model, it appeared that ligand, receptor, and activated Jaks and Stats were directly involved in nuclear events leading to specific gene activation. Therefore, the non-canonical model of IFNγ-signaling was developed, which shows large resemblance to that of steroid hormone/steroid receptor signaling (81).

In this model, IFNγ binds to the extracellular domain of the receptor and moves to the cytoplasmic side of the IFNγR1 domain, after which endocytosis of this complex is induced. The movement of Jak2 to the R1 domain results in autoactivation of the Jaks. This causes phosphorylation of R1 and eventually the recruitment and activation of Stat1α. The complex of IFNγ, the IFNγR1, Stat1α, and Jak1 and 2 is actively transported to the nucleus, a process which is under the influence of the NLS of IFNγ (14, 81, 87). The non-canonical model of IFNγ signaling formed the conceptual framework for the development of IFNγ mimetics (81).

### Intracellular routing of IFNγ and its receptor

In contrast to the numerous publications on the signal transduction and biological effects of IFNγ similar to the PDGFβ receptor, there are relatively little data available on the trafficking of the IFNγ receptor. The interferon receptor has been localized in clathrin-coated pits (CCPs), lipid rafts, and caveolae (39).

Clathrin-coated pits are specialized regions in the plasma membrane that are engaged in the efficient internalization of receptors from the cell surface. It is known that most transmembrane receptors are endocytosed via clathrin-dependent endocytosis which involves CCPs (89). Both IFNγ and its receptor were shown to be associated with CCPs (90, 91). In general, receptors are recruited into CCPs through direct binding of their cytoplasmic binding motifs to the adaptor-related protein complex 2 (AP-2).

It is suggested that other endocytotic pathways also may participate in the endocytosis of the IFNγ receptor (39). Lipid rafts are microdomains in the plasma membrane, which are enriched in certain lipids, cholesterol, and proteins, caused by redistribution within the lateral plane of cellular membranes. It is hypothesized that rafts exist in a separate phase that diffuses dynamically in a sea of poorly ordered lipids in the plasma membrane. By dynamic diffusion, proteins can be included or excluded in these rafts (91). The IFNγ receptor was shown to be present in lipid rafts. Moreover, the downstream signaling molecules Jak1 and 2 and Stat1 were also associated with lipid rafts in the plasma membrane (92–94). Caveolae are considered a subtype of lipid rafts (39). It was shown that IFNγ and its receptor also localize in caveolae, albeit to a lesser extent than in CCPs (95).

When the ligand–receptor complex is internalized via endocytosis, it enters a cellular compartment with acidic pH. Within this compartment, the ligand dissociates from the receptor. Free IFNγ can then be trafficked to the lysosome, where it is degraded. In many cells, such as fibroblasts and macrophages, the receptor eventually recycles back to the cell surface. In most cells, the intracellular pool is approximately two to four times larger when compared with the receptors expressed at the cell surface (96–100).

### Mimetic IFNγ

It was previously shown that the N-terminal side of IFNγ plays an important role in the recognition of the extracellular domain of its receptor, whereas the C-terminus is involved in endocytosis by binding to the membrane proximal region of the cytoplasmic part of the IFNγR1 subunit (101). Therefore, it is suggested that the N-terminal region of IFNγ is involved in species-specific recognition and binding, whereas the C-terminal region may be involved in binding of internalized IFNγ to the cytoplasmic receptor domain (101). This led to the development of a truncated peptide, which contains the C-terminal region of IFNγ, namely mimetic IFNγ (IFNγ_95–132 AA_) (101). It appeared that this mimetic peptide possesses similar biological activities as its full length equivalent, despite the fact that it is not able to bind to the extracellular domain of its receptor (102). It was for instance shown that mimetic IFNγ possesses IFNγ-agonistic activity without toxicity, such as the induction of MHCII expression on macrophages (101). Furthermore, mimetic IFNγ displayed antiviral activity in tissue cultures and in mice (103). In order to be able to deliver mimetic IFNγ into the cytoplasm, the peptide was modified with a single N-terminal ε-palmitoyl-lysine residue. This was shown to be suitable to allow penetration across the cell plasma membrane and subsequent cytoplasmic delivery (87, 104), but the use of this palmitoyl residue is unfavorable for *in vivo* use because it induces non-specific uptake in any cell it encounters. Similar to IFNγ, mimetic IFNγ forms a complex with IFNγR1, Stat1α, and Jak1 and 2 and translocates to the nucleus as directed by its NLS (14, 81, 87). Advantages of mimetic proteins in general are their versatility, the high biological activity and specificity, and their low toxicity (103). There are already several peptide mimetics on the market to treat a broad variety of diseases. One example is the tripeptide mimetic boceprevir for the treatment of hepatitis C viral infections (105). The therapeutic applications of mimetic IFN are nowadays explored (106).

## Antifibrotic Compounds

Although in the past decade some progress was made, the development of antifibrotic drugs for the treatment of liver cirrhosis is still in its infancy. Major steps forward in the treatment of liver diseases were made at the level of eradication of the inciting stimulus. The high successes obtained with anti-HCV drugs that effectively clear the virus and thus reverse the chronic pathology also resulted in cases in which regression of the fibrosis in the livers of these patients was ascertained (107). This contributed to the belief that liver fibrosis even in advanced state is reversible and treatable. Parallel to this, experimental therapeutic interventions directly aiming at fibrogenic key cells and processes emerged (4, 108). In 2005, Pinzani et al. showed an impressive list with all possible compounds under (pre)clinical investigation for the treatment of liver fibrosis (109). Later on, more reviews with promising antifibrotic drugs appeared (110–112). Currently 370 clinical trials are listed at http://www.clinicaltrials.gov, illustrating the upcoming incidence of clinical trials ongoing in liver cirrhosis, and showing that the pharmaceutical industry is interested to invest in drugs for the treatment of liver cirrhosis, despite the expected long-term trials to assess efficacy in this chronic, slow progressing disease. Of note, about 90% of the clinical trials are still dealing with diagnostics improvements, treatment of cirrhosis-associated complications, or with the application and evaluation of direct antiviral agents. This means that only a minority of the clinical studies really tests novel antifibrotic compounds (108). Remarkably, there are hardly any completed or ongoing clinical trials in patients with liver cirrhosis testing compounds that target PDGF and IFNγ pathways. Only in 2002, a study was started in which IFNγ1b was tested for safety and efficacy in HCV patients with liver fibrosis or cirrhosis. However, although IFNγ was well tolerated, the drug was abandoned and not approved because of lack of antifibrotic activity (113, 114). With regard to intervention at the PDGF level, several experimental approaches aiming at PDGF were described in literature. Examples include inhibition of the PDGF-B chain (115, 116), inhibition of the PDGFβ receptor expressions (117, 118), and inhibition of the PDGFβ receptor signaling (119, 120). However, none of these compounds were found in the clinical trial database on liver fibrosis or cirrhosis.

New targets for direct-acting antifibrotic compounds were identified in the past decades due to a better understanding of the pathological process at molecular level. This includes the specific roles of the different (hepatic) cell types, the mediators involved in the communication between these cells and their intracellular signaling pathways, and the unravelment of the complex process of fibrotic extracellular matrix deposition, during which matrix degrading matrix metalloproteinases (MMPs) and inhibitors of these metalloproteinases (TIMPs) parallel the enhanced production of matrix components. This basic knowledge contributed to the generation of various antifibrotic compounds that can be tested in patients with liver cirrhosis. One of the current promising ongoing clinical trials deals with the application of mesenchymal stem cells to treat liver cirrhosis (121). Mesenchymal stem cells have shown to have the ability to effectively reduce liver fibrosis and improve liver function. The effects are based on various activities of these mesenchymal stem cells. First, these stem cells can differentiate into hepatocytes, allowing the replacement of damaged hepatocytes, and they can promote regeneration of residual hepatocytes. In addition, mesenchymal stem cells also affect HSCs and possess immunomodulatory properties, all adding to their antifibrotic potential. However, some limitations need to be considered. Over the past few years, concerns have been raised about its long-term effectiveness, the potential tumorigenic risk, and the lack of standardized protocols for mesenchymal stem cell transplantation.

**Figure 2:**
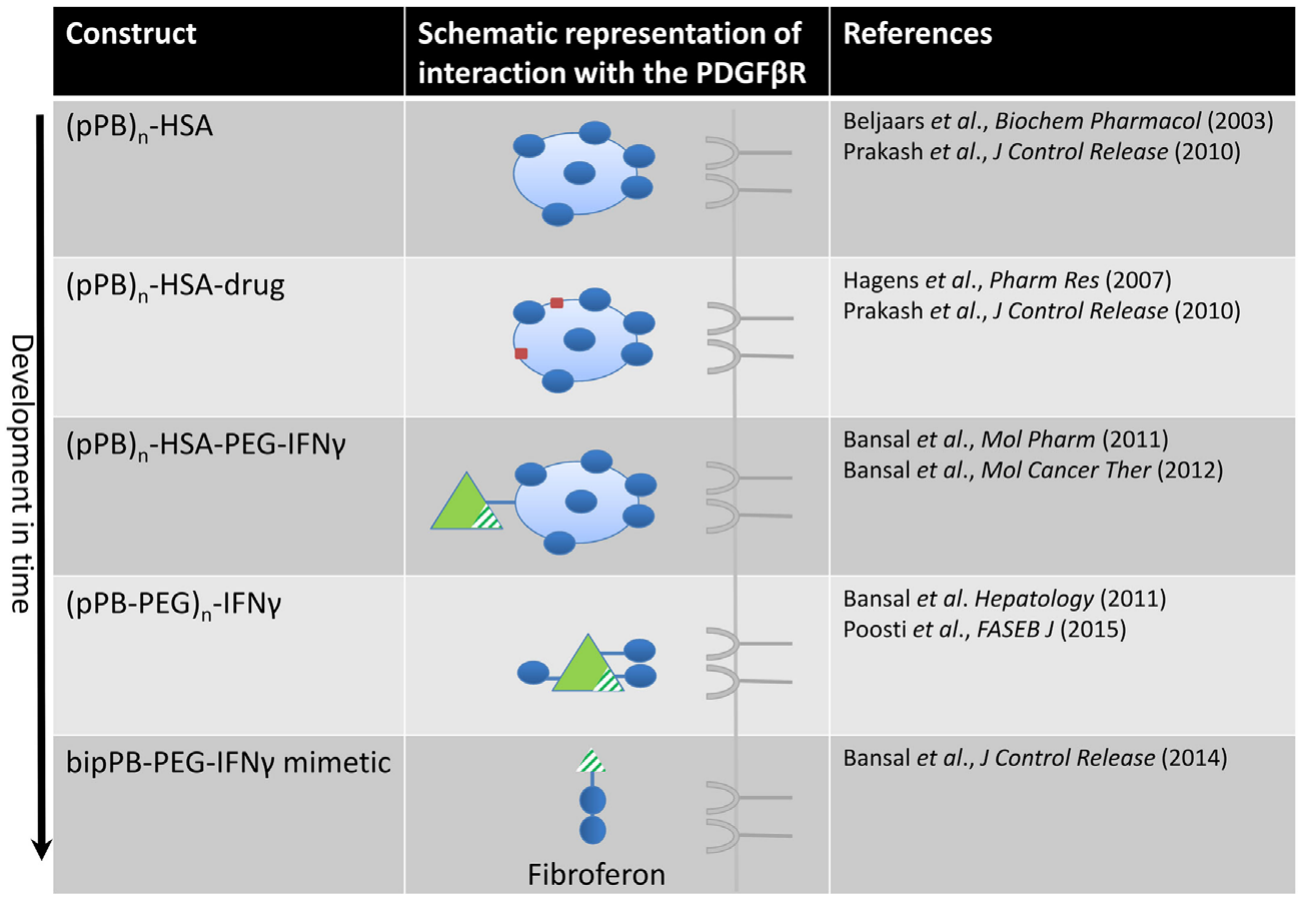
**Design of constructs directed at the PDGFβ receptor in time, finally leading to the development of Fibroferon**. The first PDGFβR-targeted product was the carrier pPB-HSA in which several (on average *n* = 10) groups of pPB were randomly attached to human serum albumin (HSA), in order to allow multivalent interaction with the PDGFR. Several other constructs, comprising a therapeutic entity (drug or protein) coupled to this carrier, were subsequently developed and tested *in vivo*. In the final step, the structure was simplified by omitting the albumin protein core and applying low-molecular-weight PEG to couple truncated IFNγ to BipPB, to create divalent interaction of truncated IFNγ with the PDGFβR.

The development of cell-specific antifibrotic drugs is another approach that seems promising (122–124). Fibroferon, based on the structure of both PDGF-BB and IFNγ, is one of the latest compounds in this context. Fibroferon is an IFNγ-derived mimetic peptide that is specifically delivered to activated HSCs and myofibroblasts. We used the highly upregulated PDGFβR as a target receptor for the HSC/myofibroblast-directed drug targeting approach in fibrosis. We were the first to be able to deliver drugs to HSCs using a series of modified proteins, and one of these drug carriers was directed at the PDGFβR (9, 125, 126). Nowadays, several groups have developed or applied cell-specific proteins to HSCs (124, 127–129), recently reviewed by Poelstra et al. (122). In the following paragraphs, we will delineate our approach and show, as depicted in Figure 2, how this has led to the development of the promising antifibrotic PDGFβR-targeted interferon, referred to as Fibroferon.

### PDGFβ receptor recognizing peptide

We designed a cyclic peptide (pPB) that mimics the binding site of PDGF-BB to its endogenous receptor. Similar to PDGF-BB itself, the arginine and isoleucine residues and their adjacent amino acids represent the PDGFβ receptor binding moieties in the pPB peptide (C*SRNLIDC*, where the C* denotes the cyclizing cysteine residues) (31). The peptide was designed in a cyclic conformation because earlier observations revealed that cyclic peptides have stronger interactions with their receptors and have increased stability in plasma as compared to their linear analogs (130, 131).

At first, a number of pPB peptides were coupled to human serum albumin (HSA) yielding the HSC-specific carrier pPB-HSA (see Figure 2) (9). The PDGFβ receptor requires a dimeric interaction for ligand binding and receptor activation (132) and therefore multiple pPB moieties (on average 10 molecules of pPB per 1 molecule HSA at different distances from each other) were necessary to obtain optimal receptor binding.

It was confirmed *in vitro* that pPB-HSA bound to the PDGFβ receptor on fibroblast cell lines and on primary rat HSCs. This binding was inhibited by PDGF-BB but not by PDGF-AA. Coupling of pPB-peptides to a macromolecule like HSA was necessary for a competitive effect with PDGF-BB, since free pPB did not displace PDGF-BB from its receptor. PDGFβR-induced effects were not found after incubation of fibroblasts with pPB-HSA. Based on these observations, it was hypothesized that coupling of pPB to a macromolecule such as HSA enabled a multivalent interaction with the PDGFβR, but no receptor activation was induced by pPB-based molecules. *In vivo*, pPB-HSA was shown to accumulate in HSCs in fibrotic human, mouse, and rat livers (9); cells expressing the highest PDGFβR. These results formed the basis for the development of HSC-selective targeting of drugs applying pPB-HSA as drug carrier and for example the antifibrotic agents IFNγ(133) and 15d-prostaglandin-J_2_ (134) were evaluated. This is particularly relevant for IFNγ, which has a therapeutic application in a wide variety of immunological, viral, and neoplastic diseases, and where high percentages, up to 100%, of patients suffer from general side effects such as headache, malaise, and fever (44). A fundamental question is however whether a cytokine can be delivered to another target receptor, without losing the biological effects elicited by its endogenous receptor.

### pPB-based delivery of IFNγ to HSCs for the treatment of liver fibrosis

We further developed HSC-selective compounds containing the cyclic peptide pPB and covalently conjugated this peptide to the antifibrotic compound IFNγ (see Figure 2). This led to various compounds either with (pPB-PEG-IFNγ and pPB-HSA-PEG-IFNγ) or without (pPB-IFNγ) polyethylene glycol (PEG) linker in between (24, 133). The choice for a distinct PEG-linker was based on previous experiments, showing that PEGylation of IFNγ with different sizes of PEG-molecules (5, 10, and 20 kDa) improved the pharmacokinetic profile, liver uptake, and antifibrotic effects of IFNγ, with the smallest PEG molecule as most favorable (72). Additionally, improved conformational flexibility of the pPB peptide using a PEG spacer allowed improved presentation of pPB to the PDGFβ receptor (72). Our conjugates were shown to bind to the PDGFβ receptor on fibroblasts and HSCs and inhibited their activation *in vitro*, as shown by a reduction in collagen type I and α-SMA expression. In addition, the conjugates were shown to inhibit PDGF-induced cell proliferation. A remarkable finding in these studies was that the specific binding to the PDGFβ receptor and subsequent biological effects of the pPB-modified IFNγ-constructs was not species-specific anymore in contrast to IFNγ. This was demonstrated by the absence of both binding and an antifibrotic effect of mouse IFNγ in human cell cultures, whereas mouse IFNγ conjugated to pPB did bind to human cells and showed biological activity (24, 133). It was therefore proposed that following binding to the PDGFβ receptor, the complex was internalized, degraded, and IFNγ (or its signaling moiety) was released into the cytoplasm leading to IFNγ-mediated effects (135).

The targeted constructs (pPB-PEG-IFNγ, pPB-HSA-PEG-IFNγ, and pPB-IFNγ) colocalized with desmin-positive PDGFβR-expressing HSCs in the liver. In addition, they strongly inhibited fibrogenesis and induced fibrolysis in a 2-week treatment study in 8-week-old CCl_4_ mice, as demonstrated by attenuated collagen type I and α-SMA deposition. Additionally, the balance between collagen-degrading MMP-13 and its endogenous inhibitor TIMP-1 was shown to be shifted toward a fibrolytic state, characterized by a reduced TIMP-1 expression. This effect was not seen in the animals treated with untargeted IFNγ. Furthermore, in contrast to untargeted IFNγ, the targeted IFNγ conjugates did not induce unwanted IFNγ-related side effects. In these mice treated with targeted IFNγ, systemic inflammation, hyperthermia, elevated plasma triglyceride levels, and neurotropic effects, as demonstrated by among others a reduced release of proinflammatory cytokines (IL-1β), were absent. Moreover, targeted IFNγ lowered MHC-II expression in the brain as compared to native IFNγ (24, 133), which makes this compound attractive for long-term treatment.

Redirection of IFNγ to myofibroblasts in the fibrotic liver proves to be a promising approach. We pursued this by creating a smaller, more simplified construct to facilitate clinical application (see Figure 2) (24, 133). This minimized structure combines three basic elements, i.e., IFNγ-activity, PEG, and a PDGFβR-binding peptide (106). In this truncated IFNγ structure (i.e., mimetic IFNγ), the extracellular IFNγ receptor binding sequence was removed, yet the NLS accommodating the biological effects of IFNγ was retained. In this way, interaction with the ubiquitously expressed IFNγ receptor at the membrane of the cells was prevented, leaving only an interaction with the intracellular IFNγR (136, 137). This mimetic IFNγ-structure was redirected to the PDGFβ receptor by coupling it to PDGFβR-recognizing peptides. We also minimized this entity by incorporating only two cyclic PDGFβ receptor binding peptides (BipPB) at a specific distinct distance from each other to allow dimeric ligand–receptor interaction. The PEG linker was minimized to only 2 kDa and BipPB, PEG, and mimetic IFNγ were subsequently coupled in a 1:1:1 ratio, yielding an 8.8 kDa construct (referred to as mimγ-BipPB, BOT191, or Fibroferon). Similar to pPB-PEG-IFNγ, Fibroferon markedly inhibited both early and advanced CCl_4_-induced liver fibrosis in mice and significantly reduced IFNγ-related side effects (106). The advantages of this targeted mimetic IFNγ-conjugate are the *in vivo* stability, the cell selectivity, dissociation of effects, the loss of species specificity, the minimal size, and less complexity, making it a better-defined product. This all provides Fibroferon with clear advantages above the therapeutic use of native IFNγ as an antifibrotic compound.

### pPB-based HSC-selective drug carriers for the treatment of other diseases

In addition to the demonstrated antifibrotic properties of redirected truncated IFNγ in a mouse liver fibrosis model, antifibrotic effects of pPB-PEG-IFNγ were also found in the unilateral ureteral obstruction (UUO) mouse model for renal fibrosis. In this model, pPB-PEG-IFNγ specifically accumulated in PDGFβR-overexpressing interstitial myofibroblasts in the kidney. Treatment of mice with pPB-PEG-IFNγ significantly attenuated collagen I, fibronectin, and α-SMA mRNA levels and protein expression in fibrotic kidneys similar to the liver fibrosis studies. Compared to vehicle treatment, pPB-PEG-IFNγ protected the tubular morphology, significantly attenuated interstitial T-cell infiltration and reduced the formation of lymphatic vessels, without affecting the peritubular capillary density. In addition, pPB-PEG-IFNγ reduced IFNγ-mediated side effects, as shown by reduced MHC-II mRNA expression in the brain (135), which was applied as a marker for off-target effects on macrophages.

pPB-HSA-IFNγ was also shown to be effective as antitumor agent (138) because the PDGFβ receptor is also upregulated in various types of cancer (139, 140). The PDGFβ receptor was immunohistochemically shown to be abundantly expressed in stromal cells and pericytes of neoplastic human diseases, like colorectal, pancreatic, and breast cancer (19). Stromal cells, including tumor-associated fibroblasts, endothelial cells, vascular pericytes, and infiltrating inflammatory cells, are increasingly recognized as key players in the induction of growth and progression of tumors (141, 142). Activation and migration of fibroblasts and tube formation in endothelial cells induced by fibroblasts was inhibited by pPB-HSA-IFNγ, suggesting an effect on angiogenic processes. In C26 colon carcinoma tumor-bearing mice, pPB-HSA-IFNγ specifically accumulated into PDGFβ receptor expressing tumor stromal fibroblasts and pericytes and inhibited the tumor growth. Treatment of these mice with pPB-HSA-IFNγ was associated with a significant inhibition of angiogenesis, as reflected by reduced α-SMA and CD31 stainings (138).

Other drugs were also delivered to the PDGFβ receptor using the cyclic peptide pPB as a targeting device. For example, the anticancer agent doxorubicin was coupled to pPB-HSA via an acid-sensitive hydrazine linkage for delivery to the PDGFβ receptor on stromal cells. The targeted construct was shown to significantly reduce tumor growth with high response rate as compared to the untargeted doxorubicin. Therefore, the delivery of anticancer drugs to these stromal cells, nurturing the neighboring tumor cells, is a novel and maybe promising approach to treat cancers effectively (143).

These studies suggest that the PDGFβ receptor-targeted constructs not only exert therapeutic effects in liver fibrosis (24, 106, 133) but also in other diseases associated with high PDGFβ receptor expression, such as kidney fibrosis (135) and some types of cancer (138, 143).

## Conclusion

The ever increasing knowledge on molecular mechanisms and mediators that orchestrate the progression of liver fibrosis can be used to design new therapeutic compounds against this lethal disease. The fundamental processes that underlie this disease are similar in many other fibrotic and sclerotic diseases, including stromal cancers, which broadens the scope of applications of such novel compounds. In the past decades, the key cells and key mediators that stimulate and inhibit liver fibrogenesis have been identified and we used this knowledge to create a cell-specific chimeric compound which combines the best of two important mediators: the high disease-induced receptor expression of one mediator (PDGFR) is used to deliver another mediator (IFNγ), endowed with antifibrotic activities, to the HSC, yielding Fibroferon. Further studies on the intracellular trafficking of this compound that induces the activation of the IFNγ-signaling cascade via binding to the PDGFR are in progress. This chimeric molecule might be one of the next-generation antifibrotic therapeutics. These studies also illustrate the surprising adaptability of biological compounds that are the natural inhibitors of this chronic disease.

## Conflict of Interest Statement

The authors declare that the research was conducted in the absence of any commercial or financial relationships that could be construed as a potential conflict of interest.
